# Blood and cerebrospinal fluid metallomics uncover mercury, chromium, and iron alterations in *de novo* Parkinson's disease

**DOI:** 10.1177/1877718X251367303

**Published:** 2025-09-04

**Authors:** Petr Dušek, Ranjani Ganapathy Subramanian, Tereza Serranová, Karel Šonka, Evžen Růžička, Jan Kuta

**Affiliations:** 1 Department of Neurology and Center of Clinical Neuroscience, First Faculty of Medicine, Charles University and General University Hospital in Prague, Prague, Czechia; 2 Department of Neurology, Jacobs School of Medicine and Biomedical Sciences, University at Buffalo, State University of NewYork, Buffalo, NY, USA; 3 RECETOX, Faculty of Science, Masaryk University, Brno, Czechia

**Keywords:** Parkinson's disease, metallomics, mercury, iron, chromium

## Abstract

**Background:**

Given the increasing global prevalence of Parkinson's disease (PD) and its complex etiopathogenesis, understanding the role of environmental factors is crucial. Prior investigations suggested a potential link between metal exposure and PD, yet conflicting results emerged.

**Objective:**

To identify differences in metal concentrations in whole blood and cerebrospinal fluid (CSF) in PD patients compared to controls.

**Methods:**

The study involved an untreated de novo PD patient cohort from a single-center (n = 102, 38% females, mean age 59.5 (SD 12.5)) and a group of controls with comparable age and sex distribution (n = 127, 35% females, mean age 57.5 (SD 12.4)). Whole blood in all participants and CSF samples in a subgroup (n = 57/55 PD/controls) were collected and concentrations of V, Cr, Mn, Fe, Co, Ni, Cu, Zn, As, Se, Mo, Cd, Sn, Hg, and Pb, were determined through inductively coupled plasma mass spectrometry.

**Results:**

PD patients exhibited higher concentrations of Hg in both blood and CSF (p = 0.003). Cr concentrations were lower in both blood (p = 0.004) and CSF (p < 0.001) of PD patients. Altered Fe metabolism was evident, with higher blood (p = 0.001) and lower CSF (p = 0.002) Fe concentrations. Other metal alterations in PD included higher Zn (p = 0.008) in blood and lower Co (p < 0.001), Mn (p = 0.006), V (p = 0.009), and Ni (p < 0.001) in CSF.

**Conclusions:**

The findings highlight abnormalities in metal concentrations in biofluids associated with PD, particularly regarding Hg, Cr, and Fe which exhibited alterations in blood and CSF. These findings suggest metal dysregulation in PD, particularly Hg, Cr, and Fe, with potential implications for understanding PD pathogenesis.

## Plain language summary

Parkinson's disease (PD) is increasing globally, and environmental factors, including metal exposure, may play a role. This study investigated metal concentrations in blood and cerebrospinal fluid of untreated newly diagnosed PD patients compared to controls. Blood samples from all participants and cerebrospinal fluid from a subset were analyzed for 15 metals using inductively coupled plasma mass spectrometry. PD patients had higher mercury levels in both blood and cerebrospinal fluid, lower chromium levels in blood and cerebrospinal fluid, and altered iron metabolism, with higher blood and lower cerebrospinal fluid concentrations. Other significant metal alterations in PD included higher blood zinc and lower cerebrospinal fluid cobalt, manganese, vanadium, and nickel. These findings suggest metal dysregulation in PD, particularly mercury, chromium, and iron, with potential implications for understanding PD pathogenesis.

## Introduction

Parkinson disease (PD) is the second most frequent neurodegenerative disorder affecting more than 6 million people globally.^1^ Among neurological disorders examined in the Global Burden of Disease, Injuries, and Risk Factors Study, PD was the fastest growing in prevalence, disability, and deaths due to which the term “Parkinson pandemic” was coined. The prevalence of PD increased by 74% from 1990 to 2016 which is not solely due to population aging since age-standardized prevalence increased by 22%.^2^ For example, in Finland, incidence of early-onset PD (with age at onset < 55 years) increased 1.7x from 1995 to 2006.^3^ In the USA, the total annual economic burden of PD was estimated to be $52 billion^4^ and is expected to rise in the next decades. Therefore, it is a great priority for global healthcare to better understand etiopathogenetic factors and find means of prevention or causal treatment of PD.

Currently, etiology of PD and reasons for its increasing incidence are unknown. Genetic factors explain approximately 20% of PD incidence and they are unlikely to be responsible for growing prevalence of PD.^5^ Therefore, the attention of PD researchers is shifting towards environmental factors^6^ and the potential of several xenobiotics^7^ and viral infections^8^ to trigger neurodegeneration is studied. There is strong epidemiological evidence linking PD with the exposure of pesticides such as rotenone, paraquat, or maneb.^9^ Other studies have indicated increased risk of PD associated with long-term exposure to organic solvents.^10^

It was also suggested that exposure to metals may be related to the development of PD.^11,12^ Human exposure to metals has increased in the last decades through several metal sources present in the environment, including geogenic, industrial, agricultural, pharmaceutical, and home effluents.^13,14^ The neurotoxicity of heavy metals with potential link to neurodegeneration may be mediated through several mechanisms including oxidative stress, impairment in mitochondrial function, endoplasmic reticulum stress, alpha-synuclein and other protein misfolding, activation of microglia, and apoptosis. Additionally, metals are known to accumulate in basal ganglia where they may aggravate the progression of alpha-synucleinopathy-related neuropathological changes and hence clinical symptoms and theoretically can be targeted by chelators.^12,15^

Previous studies suggested the role of various metals in the etiopathogenesis of PD, specifically mercury,^16^ copper,^17^ manganese,^18^ arsenic,^19^ and lead.^20^ Clusters of increased PD prevalence were reported in regions with higher metal exposure; e.g., parts of the northwestern and midwestern U.S. previously involved in industrial manufacturing and coal mining partially overlap with regionally increased incidence of PD, also called the “PD Belt”.^21^ Exposure of metals in vivo can be studied by measuring their concentration in body fluids and tissues of which blood is the easiest to sample. Importantly, several toxic metals, e.g., mercury, cadmium or lead accumulate in red blood cells.^22–24^ It may be thus advantageous to measure their levels in the whole blood in order to properly assess the exposure. Several studies comparing concentration of metals in various tissues in PD and healthy controls were performed with conflicting results.^25–31^ Among others, small sample size, contamination during sampling, different disease stages or PD subtypes, and regional variations in metal exposure might have contributed to these disparities.^32^ Given the great potential of possible preventive and therapeutic measures, the role of metal exposure in PD should be ascertained in more detail. In order to examine the potential contribution of metal exposure to PD pathogenesis it is important to study the prodromal or early symptomatic disease stages. However, most studies performed to date analyzed more advanced disease stages, where changes in metal concentrations may be affected by metabolic alterations secondary to neurodegeneration, chronic dopaminergic treatment, or lifestyle changes due to disability. Thus, further metallomic studies in early-stage untreated patients are needed.

Therefore, the aim of this study was to examine alterations in concentrations of potentially toxic metals in blood and cerebrospinal fluid (CSF) in a homogeneous, phenotypically well-characterized cohort of untreated de novo PD patients from Czechia, central Europe as compared to age-matched controls.

## Methods

### Study population

This study is part of a longitudinal project “biomarkers in PD (BIO-PD)” aimed at collecting a large representative sample of de novo PD patients.^33^ For inclusion, PD patients had to be diagnosed based on the Movement Disorder Society clinical diagnostic criteria for PD^34^ including abnormal dopamine transporter imaging using ^123^I-Ioflupane (DaTSCAN™). Blood and optionally also CSF sampling had to be performed before dopaminergic treatment initiation.

Three groups of controls were used: 1) healthy volunteers recruited from the general community through advertisements, who were neurologically examined to exclude parkinsonism and cognitive impairment; 2) symptomatic neurological controls who had diagnostic lumbar puncture with normal finding (defined as CSF white blood cell count ≤15/μL and CSF protein concentration ≤1 g/L) and who were diagnosed with non-inflammatory, non-neurodegenerative conditions; 3) spinal anesthesia subjects undergoing elective surgery for urological disorders without a history or signs of neurodegenerative disorder. Blood samples were acquired from all groups while CSF samples were taken only in groups 2 and 3. The inclusion of clinical control groups, who underwent lumbar puncture as part of their clinical management, was necessary due to the practical and ethical challenges of obtaining CSF from healthy volunteers. Clinical controls had various disorders including functional movement disorder, narcolepsy, idiopathic hypersomnia, headache, nerve root compression in group 2, and prostatic hyperplasia, hydrocele, urethral stricture, and phimosis in group 3. Heterogeneous control samples from patients with various neurological and non-neurological disorders were included to minimize potential bias related to altered metal homeostasis in any single condition.

Exclusion criteria for all groups were age ≤30 years, anemia defined as blood hemoglobin concentration <120/130 g/L in females/males, and active oncologic illness, since both these conditions may affect metal concentrations.^35–37^ For CSF analysis, an additional exclusion criterion was contamination of blood defined as CSF erythrocyte count ≥ 500/µL.

PD patients underwent a comprehensive examination according to the protocol, which included structured interview, vital signs, cognitive screening using the Montreal Cognitive Assessment (MoCA),^38^ motor assessment using part III of the Movement Disorder Society-Unified Parkinson's Disease Rating Scale (MDS-UPDRS),^39^ olfactory testing with the University of Pennsylvania Smell Identification Test (UPSIT),^40^ and evaluation of autonomic symptoms using the Scale for Outcomes in Parkinson's disease for Autonomic symptoms (SCOPA-AUT).^41^ For all control participants, medical charts including their clinical history and current medication were available; the information regarding history of diabetes, liver and renal disorders were retrieved. Control participants had vital signs examined and filled out a questionnaire regarding their neurologic and cognitive status, smoking, and alcohol use.

This study was approved by the ethics committee of the General University Hospital in Prague. Participants with PD were informed about the study and provided written informed consent; participants from the control group provided samples for biomarker research as per universal biobank informed consent.

### Collection of samples

Venous blood and CSF samples were drawn in the morning after an overnight fast. The collection site for venipuncture and spinal tap was cleaned with alcohol disinfectant. Blood was collected into BD Vacutainer^®^ K_2_EDTA 10 ml tubes and gently inverted 8–10 times immediately after collection to ensure proper mixing with the anticoagulant. Lumbar punctures were performed using 22 G atraumatic needles in PD patients and neurologic controls and by 25 G needles in patients undergoing spinal anesthesia. Approximately 3 ml of CSF was drawn into polypropylene tubes that were prewashed with ultraclean 5% HNO_3_ to eliminate background metal contamination. Whole blood and uncentrifuged CSF samples were transferred to the biobank on ice and immediately frozen at −80°C within two hours after collection without further processing until the analysis. Whole blood was analyzed to enhance the accuracy of detecting several toxic metals, including Hg, Pb, and Cd, which are known to accumulate preferentially in erythrocytes and their plasmatic concentration underestimates total metal burden. Blood and CSF samples were also sent for routine clinical examination including blood count, serum creatinine, and liver function tests. These biochemical analyses were performed in the laboratory of the Institute of Medical Biochemistry and Laboratory Diagnostics of the General University Hospital in Prague on automated analyzers.

### Determination of trace elements in whole blood and cerebrospinal fluid

An extended basic set of toxic and biogenic metals, Vanadium (V), Chromium (Cr), Manganese (Mn), Iron (Fe), Cobalt (Co), Nickel (Ni), Copper (Cu), Zinc (Zn), Arsenic (As), Selenium (Se), Molybdenum (Mo), Cadmium (Cd), Tin (Sn), Mercury (Hg), and Lead (Pb), was examined. These 15 metals were selected based on the customs and long-standing expertise of the metallomics laboratory conducting the analysis. Their concentrations in samples of whole blood and cerebrospinal fluid (CSF) were determined using inductively coupled plasma mass spectrometry (ICP-MS, Agilent 7700x, Agilent Technologies). Prior to processing, samples were removed from the freezer, allowed to thaw completely at room temperature, and processed immediately thereafter. The samples were diluted 10 times (for blood) and 5 times (for CSF) prior analysis with a solution containing deionized water, a nonionic surfactant Triton X-100 (0.04%), ammonia (1%), butanol (2%), EDTA (0.04%), and appropriate internal standards for ICP-MS (20 ng/ml of Sc, Ge, In, Lu, and Bi).

The method's validation included the analysis of samples spiked with a known amount of analytes and the analysis of certified reference materials (SERO AS, Norway): Seronorm™ Trace Elements Serum L-1, Seronorm™ Trace Elements Serum L-2, Seronorm™ Trace Elements Whole Blood L-1, and Seronorm™ Trace Elements Whole Blood L-2. The ICP-MS measurements demonstrated recoveries typically ranging from 85% to 115% for both the spiked samples and the certified reference materials. Laboratory blanks (diluted aliquot of deionized water) and certified reference materials were consistently analyzed through the analytical sequence with frequency one blank and one reference material for every 10–15 samples. Field/transport blanks (empty polypropylene tubes subsequently filled with deionized water) were also analyzed and no significant differences between laboratory and field blanks were found.

### Data analysis

For descriptive purposes, concentrations of trace elements below the limit of detection (LOD) were substituted with the values equal to LOD divided by two. The distribution of metal concentration values was checked visually using frequency histograms and QQ plots. For metal concentrations exhibiting a heavy-tailed distribution, a log_10_ transformation was applied.

For all metals in which any censored data (i.e., concentration values below LOD) were present in either group, comparisons between PD and controls were performed using the Peto-Prentice test. This non-parametric method accommodates left-censored data without the need for substitution. The test was implemented in R using the cendiff() function from the NADA package. For other between-group comparisons, analysis of covariance (ANCOVA) with group and sex as explanatory variables and age as a covariate was used. Cramer's phi (ϕ) and partial eta squared (η²) were used as effect size measures for the Peto-Prentice test and ANCOVA, respectively.

Additionally, for metals with left-censored concentration values, we performed a sensitivity analysis accounting for age and sex as covariates. Given the presence of censored data, non-normality, and heteroscedasticity, we employed Censored Quantile Regression (CQR) to estimate the effects of group, age, and sex across different parts of the conditional distribution of metal concentrations at quantiles τ = 0.25 (lower quartile), 0.5 (median), and 0.75 (upper quartile). CQR extends standard quantile regression by properly handling left-censored data, ensuring that censored observations influence lower quantiles more than higher ones while preventing bias from mean imputation.^42^ CQR models were estimated using the Portnoy method (quantreg::crq, method = “Portnoy”) in R.

The Benjamini-Hochberg method was applied to control the false discovery rate (FDR) at 5%. The correction was applied separately for analyses of metal concentrations in blood and CSF, treating each as distinct family of hypotheses. Results were considered statistically significant if the Benjamini–Hochberg-adjusted p-value (q-value) was below 0.05. For transparency, uncorrected p-values are reported throughout the manuscript. Associations between metal concentrations and clinical and demographic parameters were assessed using Spearman correlation coefficient. Binary categorical variables were analyzed using Fisher's exact test. P-value below 0.05 was considered statistically significant.

IBM SPSS statistics version 25 (IBM, Armonk, NY, USA) was used for statistical analysis; graphs were plotted using Graphpad Prism version 9.0 (Graphpad software, San Diego, CA, USA). The Peto-Prentice and CQR analyses were conducted in R v4.4.3.

## Results

### Participant characteristics

**Figure 1.**
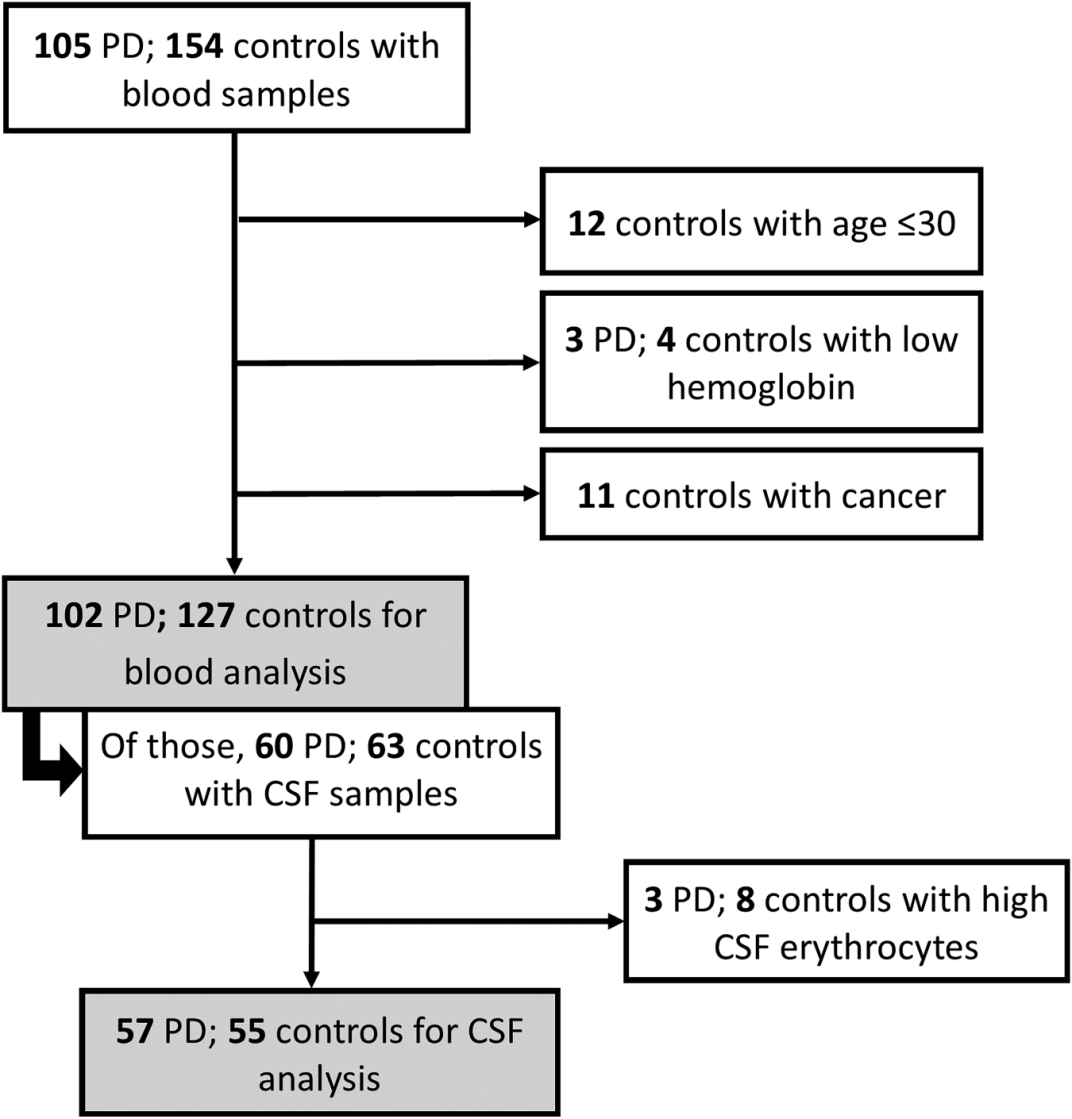
Flowchart of patient inclusion. PD: Parkinson's disease; CSF: cerebrospinal fluid.

Of the 105 PD and 154 controls enrolled between 2015 and 2022 who had blood samples available, 3 PD and 27 controls did not meet the inclusion criteria. Thus, 102 PD and 127 control (Group 1: n = 64, Group 2: n = 39, Group 3: n = 24) blood samples were available for analysis. Of those, 60 PD and 63 controls had CSF available while 3 PD and 8 control CSF samples did not meet the inclusion criteria yielding 57 PD and 55 control (Group 2: n = 35, Group 3: n = 20) CSF samples for analysis (Figure 1).

Demographic and clinical characteristics of PD and control groups are reported in Table 1. Both groups had comparable sex distribution, age, BMI, alcohol consumption, diabetes prevalence, red blood count and blood hemoglobin concentration. None of the participants had a history of major liver or renal dysfunction such as cirrhosis, chronic hepatitis, or chronic nephritis. Slightly elevated creatinine levels were observed in five (5%) PD patients and seven (6%) controls, and a slight increase in liver function test results was noted in six (6%) PD patients and seven (6%) controls. There were more active smokers among the controls compared to the PD group (p = 0.003). The subgroup for CSF analysis was representative of the total group although it was slightly younger, and PD patients had higher red blood count and hemoglobin levels compared to controls (p = 0.002).

**Table 1.** Demographic and clinical information for study participants.

	blood			CSF		
	PD (n = 102)	Controls (n = 127)	p	PD (n = 57)	Controls (n = 55)	p
Sex [n] M/F^a^	63/39	83/44	0.574	39/18	33/22	0.431
Age [years]^b^	59.5 (12.5)	57.5 (12.4)	0.233	56.3 (11.9)	53.9 (14.1)	0.328
Body mass index^b^	27.5 (3.8)	28.3 (5.3)	0.219	26.9 (2.9)	28.4 (5.8)	0.072
Alcohol [standard drinks per month]^b^	18.3 (21.6)	21.9 (23.2)	0.279	19.6 (24.1)	15.6 (13.1)	0.528
Current smokers [n] (%)^a^	6 (5.9%)	25 (19.7%)	**0**.**003**	5 (8.8%)	16 (29.6%)	**0**.**007**
Blood hemoglobin [g/L]^c^	148.8 (12.8)	145.9 (12.3)	0.069	150.1 (12.6)	142.9 (9.4)	**0**.**002**
Red blood count [x10^12^/L]^c^	4.9 (0.5)	4.8 (0.5)	0.101	5.0 (0.4)	4.6 (0.5)	**0**.**002**
Creatinine [μmol/L]^c^	79.0 (13.9)	78.4 (15.8)	0.929	80.5 (12.5)	77.5 (16.1)	0.550
Alanine transaminase [µkat/L]^c^	0.43 (0.17)	0.46 (0.24)	0.417	0.43 (0.19)	0.43 (0.22)	0.904
Aspartate transaminase [µkat/L]^c^	0.42 (0.10)	0.42 (0.17)	0.881	0.41 (0.09)	0.36 (0.12)	0.036
Diabetes [n] (%)^a^	9 (8.8%)	16 (12.6%)	0.399	7 (12.3%)	9 (16.4%)	0.597
Disease duration [years]	1.9 (1.8)	n.a.	n.a.	1.8 (1.8)	n.a.	n.a.
MDS-UPDRS III	29.8 (11.7)	n.a.	n.a.	28.4 (11.8)	n.a.	n.a.
MoCA	25.1 (3.1)	n.a.	n.a.	25.2 (3.3)	n.a.	n.a.
UPSIT	23.3 (6.6)	n.a.	n.a.	24.7 (6.3)	n.a.	n.a.
SCOPA-AUT	8.7 (5.2)	n.a.	n.a.	8.1 (5.0)	n.a.	n.a.

Values are reported as mean (standard deviation) unless otherwise specified. Statistically significant p-values are indicated in **bold**.

^a^ analyzed by Fisher's exact test; ^b^ analyzed by Student's t-test; ^c^ analyzed by ANCOVA adjusted for age and sex.

PD: Parkinson disease; CSF: cerebrospinal fluid; M: male; F: female; n.a.: not available; MDS-UPDRS: Movement Disorder Society-Unified Parkinson's Disease Rating Scale; MoCA: Montreal Cognitive Assessment; SCOPA-AUT: Scale for Outcomes in Parkinson's disease for Autonomic symptoms; UPSIT: University of Pennsylvania Smell Identification Test.

### Metal concentrations in blood and CSF

**Figure 2.**
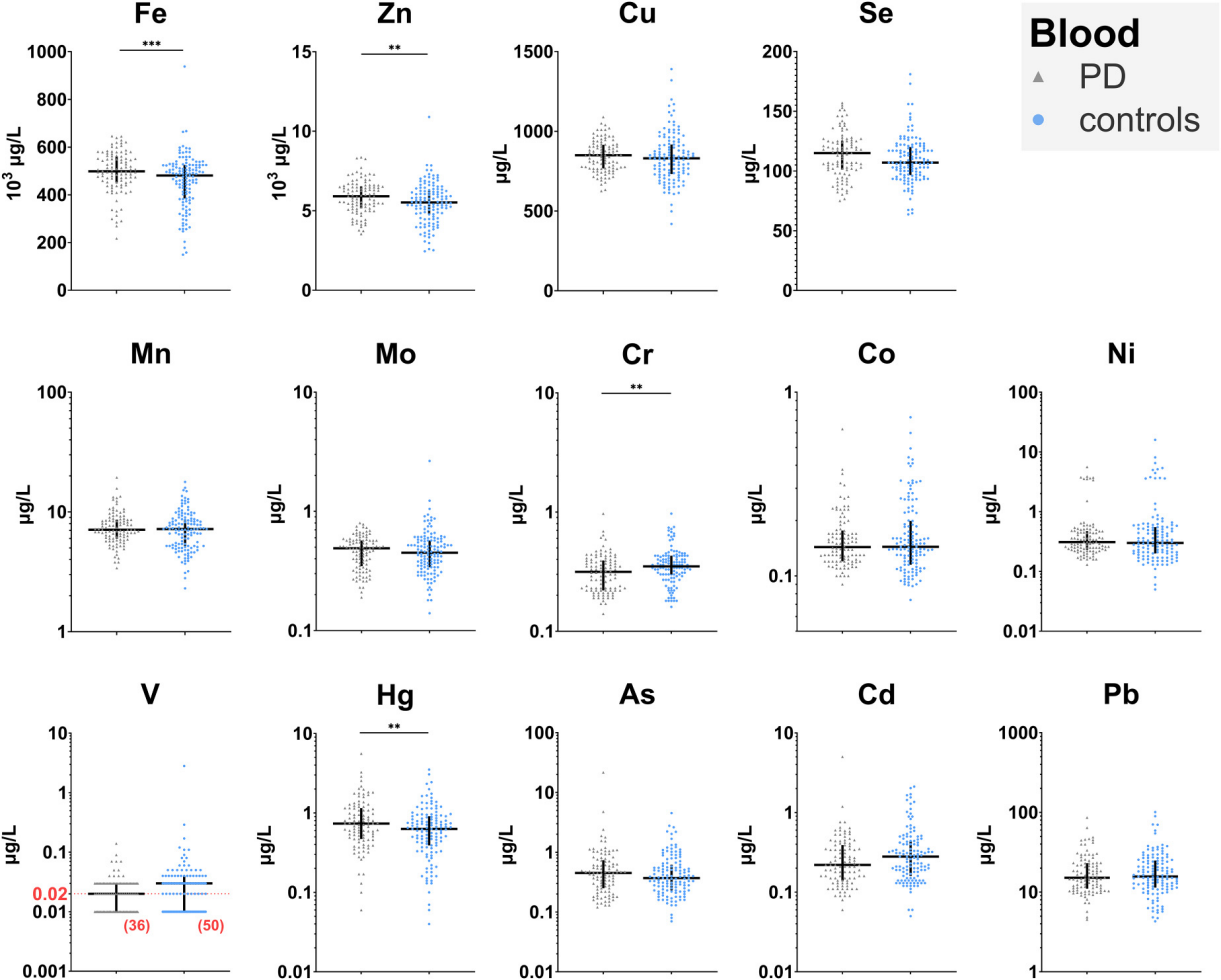
Dot graphs of blood metal concentrations. In each metal graph, PD is shown on the left (grey triangles) and controls on the right (blue circles). Except for Fe, Zn, Cu, and Se, values are shown on a logarithmic scale. When relevant, the detection limit is shown in red color and marked by a dotted line in the graph; number of samples with concentration below the detection limit is reported in brackets. Horizontal lines in dot graphs denote medians; vertical lines denote interquartile range. Significant between-group differences are marked as follows: * < 0.05, ** < 0.01, *** < 0.001 based on ANCOVA with group and sex as explanatory variables and age as a covariate.

**Figure 3.**
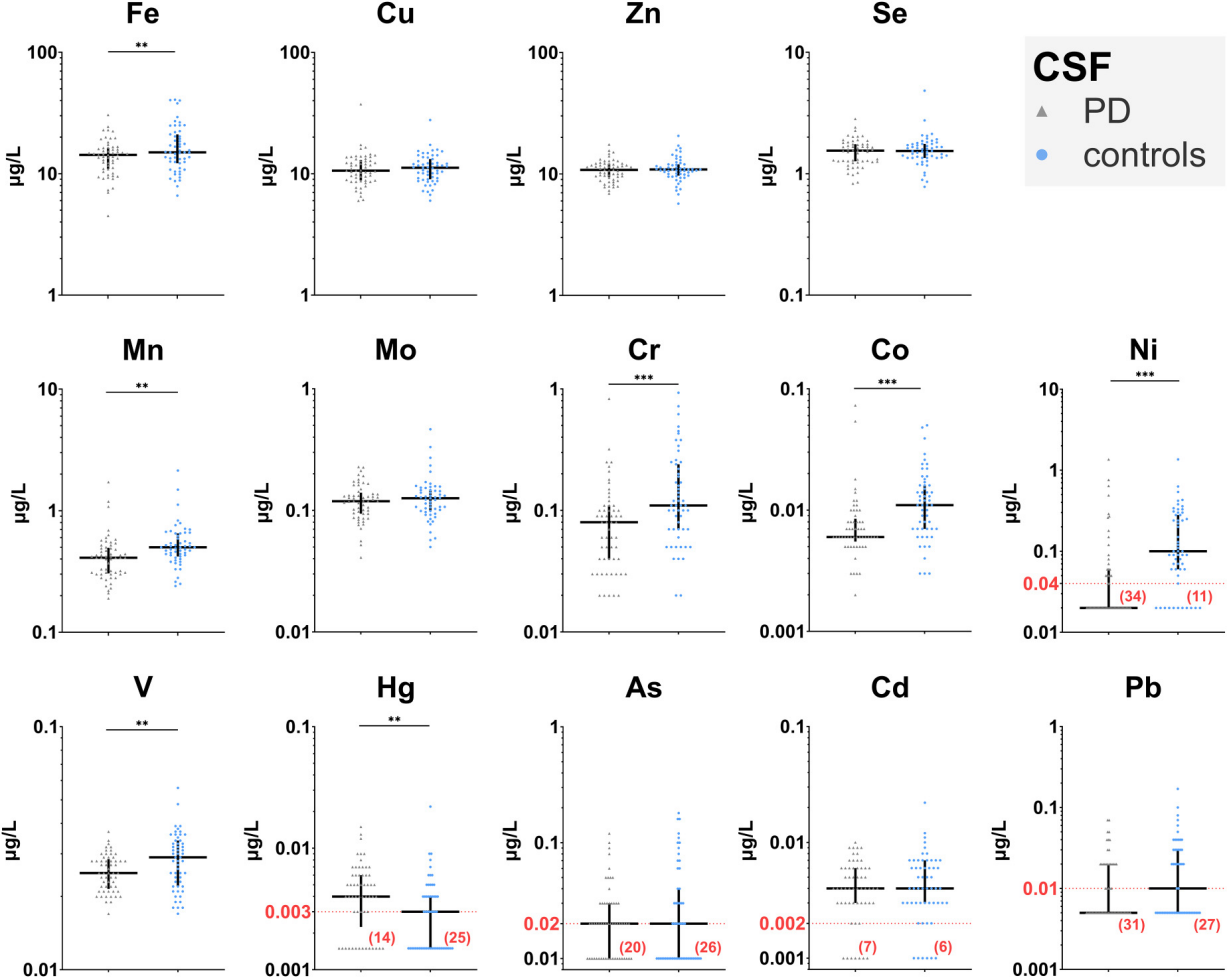
Dot graphs of CSF metal concentrations. In each metal graph, PD is shown on the left (grey triangles) and controls on the right (blue circles). Values are shown on a logarithmic scale. When relevant, the detection limit is shown in red color and marked by a dotted line in the graph; number of samples with concentration below the detection limit is reported in brackets. Horizontal lines in dot graphs denote medians; vertical lines denote interquartile range. Significant between-group differences are marked as follows: * < 0.05, ** < 0.01, *** < 0.001 based on ANCOVA with group and sex as explanatory variables and age as a covariate, except for Ni and Hg, which were analyzed using the Peto-Prentice test.

The concentrations of Sn in blood and CSF were below LOD in more than 50% samples and Sn levels were thus not further analyzed. Distributions of metal concentrations in blood and CSF are depicted in Figures 2 and 3, respectively. Blood levels of Fe, Zn, Cu, and Se displayed a normal distribution and were analyzed using ANCOVA models without any transformations. Some concentrations of blood V (35% in PD and 39% in controls) and CSF Ni (59% in PD and 20% in controls), Hg (24% in PD and 45% in controls), As (35% in PD and 47% in controls), Cd (13% in PD and 11% in controls), and Pb (54% in PD and 49% in controls) were below LOD and these metals were analyzed by the Peto-Prentice test. All other metal levels in blood and CSF exhibited a heavy-tailed distribution and were analyzed by ANCOVA models following a log_10_ transformation.

There was a significant effect of sex on several metal concentrations. In the blood, Fe, Zn, and Pb were more abundant in males while Cu, Mo, Co, and Ni were more abundant in females. In CSF, Fe and Zn were more abundant in males. No significant group-by-sex interaction was found for any of the metals with sex-related differences.

Compared to controls, PD patients had higher blood concentrations of Fe (p = 0.001, η²=0.046), Zn (p = 0.008, η²=0.031), and Hg (p = 0.003, η²=0.038) and lower blood concentration of Cr (p = 0.004, η²=0.044); see Table 2. Sensitivity analysis confirmed that between-group differences in blood Fe (p < 0.001), Zn (p = 0.004), Hg (p < 0.001), and Cr (p = 0.008) concentrations remained significant when hemoglobin concentration was used as an additional covariate. The effect of hemoglobin concentration was significant for Fe and Zn levels but not for Hg and Cr levels. There was a significant interaction between group and sex for blood Cd concentration (p = 0.006); it was lower in PD males (p < 0.001, η²=0.088) while there was no significant between-group difference in females. After excluding smokers, the difference in blood Cd concentration between PD and control males was not significant (p = 0.071).

**Table 2.** Comparison of blood and CSF metal concentrations in PD and controls.

blood				CSF			
Metal [µg/L]	PD	controls	uncorrected p	Metal [ng/mL]	PD	controls	uncorrected p
Fe (x10^3^)^a^	M 516.7 (84.2)	M 468.3 (96.7)	**0.001** ^s,a−^	Fe	M 14.8 (14.4; 12.3–16.6)	M 20.8 (17.6; 15.0–25.0)	**0.002^s^** ^,a+^
	F 461.6 (82.9)	F 432.3 (135.6)			F 13.2 (12.7; 9.1–15.8)	F 12.6 (12.5; 9.9–14.6)	
Zn (x10^3^)^a^	M 6.05 (1.07)	M 5.61 (1.11)	**0.008** ^s,a−^	Zn	M 11.3 (11.3; 9.8–12.4)	M 11.8 (11.1; 10.4–13.0)	0.128^s,a+^
	F 5.55 (0.93)	F 5.24 (1.51)			F 10.0 (9.6; 8.2–11.5)	F 10.1 (10.4; 8.9–10.9)	
Cu^a^	M 807.8 (86.2)	M 779.8 (113.3)	0.841^s^	Cu	11.4 (10.6; 8.8–12.8)	11.4 (11.2; 9.0–13.2)	0.507^a+^
	F 904.8 (87.7)	F 944.6 (164.4)					
Se^a^	113.8 (19.1)	109.2 (19.1)	0.055^a−^	Se	1.54 (1.55; 1.27–1.75)	2.10 (1.54; 1.35–1.76)	0.080^a+^
Mn	7.64 (7.10; 6.00–8.60)	7.41 (7.20; 5.20–8.70)	0.111^a−^	Mn	0.447 (0.410; 0.310–0.490)	0.559 (0.500; 0.420–0.650)	**0**.**006**
Mo	M 0.44 (0.44; 0.32–0.57)	M 0.48 (0.43; 0.34–0.53)	0.999^s^	Mo	0.123 (0.119; 0.094–0.137)	0.134 (0.126; 0.099–0.147)	0.393
	F 0.52 (0.54; 0.43–0.58)	F 0.51 (0.52; 0.39–0.61)					
Cr	0.33 (0.32; 0.22–0.39)	0.37 (0.35; 0.30–0.43)	**0.004** ^a+^	Cr	0.100 (0.080; 0.040–0.110)	0.182 (0.110; 0.070–0.240)	**<0**.**001**
Co	M 0.15 (0.14; 0.12–0.17)	M 0.16 (0.13; 0.11–0.16)	0.345^s,a−^	Co	0.009 (0.006; 0.006–0.008)	0.014 (0.011; 0.007–0.016)	**<0.001** ^a+^
	F 0.18 (0.15; 0.13–0.21)	F 0.23 (0.17; 0.15–0.32)					
V^b^	0.03 (0.02; 0.01–0.03)	0.07 (0.02; 0.01–0.03)	0.196	V	0.025 (0.025; 0.022–0.028)	0.029 (0.029; 0.022–0.034)	**0**.**009**
Ni	M 0.54 (0.28; 0.22–0.45)	M 0.77 (0.27; 0.17–0.49)	0.901^s^	Ni^b^	0.092 (0.000; 0.000–0.006)	0.181 (0.100; 0.060–0.280)	**<0**.**001**
	F 0.75 (0.38; 0.24–0.52)	F 1.01 (0.42; 0.26–0.58)					
As	0.80 (0.45; 0.25–0.73)	0.57 (0.37; 0.23–0.62)	0.265	As^b^	0.023 (0.020; 0.000–0.030)	0.032 (0.020; 0.000–0.040)	0.942
Cd	M 0.22 (0.18; 0.13–0.26)	M 0.41 (0.26; 0.15–0.43)	**M** **<** **0.001** F 0.343	Cd^b^	0.004 (0.004; 0.003–0.006)	0.005 (0.004; 0.003–0.007)	0.716
	F 0.50 (0.39; 0.22–0.55)	F 0.36 (0.30; 0.19–0.44)					
Hg	0.92 (0.74; 0.47–1.15)	0.73 (0.63; 0.39–0.91)	**0.003**	Hg^b^	0.004 (0.004; 0.003–0.006)	0.003 (0.003; 0.000–0.004)	**0**.**003**
Pb	M 21.2 (18.4; 12.7–25.2)	M 23.7 (19.3; 14.3–27.6)	0.787^s,a+^	Pb^b^	0.012 (0.000; 0.000–0.020)	0.021 (0.010; 0.000–0.030)	0.223
	F 16.4 (12.0; 10.2–16.2)	F 13.9 (11.1; 7.8–15.4)					

^a^ values are reported as mean (standard deviation); statistical analysis performed using ANCOVA with group and sex as explanatory variables and age as a covariate on non-transformed data.

^b^ values are reported as mean (median; interquartile range) calculated from the substituted dataset; statistical analysis performed using the Peto-Prentice test.

All other values are reported as mean (median; interquartile range); statistical analysis performed using ANCOVA with group and sex as explanatory variables and age as a covariate on log10-transformed data.

^s^ significant effect of sex; in case of significant sex effect, values are reported for males and females separately.

^a+^ significant positive effect of age.

^a−^ significant negative effect of age.

P-values significant after multiplicity correction using the Benjamini-Hochberg method (adjusted p-value (q-value) < 0.05) are shown in **bold**.

PD: Parkinson disease; CSF: cerebrospinal fluid; M: male; F: female.

In the CSF, PD patients had lower concentrations of Fe (p = 0.002, η²=0.088), Mn (p = 0.006, η²=0.068), Cr (p < 0.001, η²=0.103), Co (p < 0.001, η²=0.107), Ni (p < 0.001, ϕ=0.437) and V (p = 0.009, η²=0.062) and higher concentration of Hg (p = 0.003, ϕ=0.286).

The results of the sensitivity analysis using CQR for metals with left-censored concentration values were consistent with the findings of the Peto-Prentice test, confirming higher CSF Hg and lower CSF Ni in PD patients across all estimable quantiles. Additionally, CQR indicated lower blood V and lower CSF Pb at higher quantiles in the PD group. Sex differences were also evident, with higher CSF Ni and Hg and higher blood V observed in men compared to women. The full CQR results are summarized in Supplemental Table 1.

### Correlations between metal concentrations, lifestyle factors, and clinical variables

Correlations between metal concentrations, demographic and clinical variables for each group and biofluid are shown in Supplemental Tables 2–5. Notably, there was a positive effect of age on blood concentration of Pb and CSF concentrations of Fe, Zn, Cu, and Se. Smoking (number of cigarettes per day) was positively associated with blood Cd while alcohol intake (mean number of standard drinks per month) was positively associated with blood V, and Pb and negatively associated with blood Mo. Diabetes was associated with higher Fe, Cu, and Zn levels in CSF. The associations between metal concentrations and the severity of motor, cognitive, autonomic, and olfactory dysfunction were largely insignificant, except for a positive association between the SCOPA-AUT score and Fe, Cu, and Se concentrations in the CSF. After adjusting for age, only the association with Se remained significant (p < 0.001).

To provide insight into metal kinetics, such as retention in peripheral compartments, blood-brain-barrier function and clearance from the CNS compartment, we calculated both correlations and ratios between blood and CSF concentrations of metals. Correlations between blood and CSF concentrations are shown in Supplemental Table 6. In both groups, blood and CSF concentrations of Cr, As, Mo, and Hg were significantly correlated, suggesting a degree of equilibrium between blood and CSF compartments for these metals. Blood/CSF concentration ratios are reported in Supplemental Table 7. Lower blood/CSF ratios of Cr, Co, V, and Ni reflect lower CSF concentrations in PD. In contrast, higher blood/CSF ratio and lack of correlation for Fe in PD reflect elevated blood and reduced CSF concentrations, suggesting independent regulation of Fe levels across compartments in this group.

## Discussion

We performed profiling of metal concentrations in the whole blood and CSF in a relatively large homogenous group of de novo untreated PD patients as compared to controls and found increased Hg and decreased Cr in both compartments in the PD group. Additionally, PD patients had higher concentrations of Fe and Zn in blood and lower concentrations of Fe, Mn, Co, V, and Ni in CSF compared to controls.

Several previous studies examined metal concentrations in various tissues in PD patients with inconsistent findings. High heterogeneity of patterns of CSF elemental concentrations in PD was confirmed in a multicentric study where strong center-specific bias was observed.^25^ This bias may be related to differences in disease stage, sampling procedure, geographical and environmental factors, local dietary habits, with an additional effect of small numbers of patients and/or controls. A recent meta-analysis exploring the association between metal exposure and PD risk reported lower Fe, Zn, and Cu levels in serum and higher Mg levels in CSF in PD patients compared to controls.^32^

Since we analyzed whole-blood, our results more accurately reflect the total burden of toxic metals that accumulate in erythrocytes and are not directly comparable to most previous studies, which analyzed serum or plasma metal concentrations. Previous studies also employed older participants with a mean age ranging from 65 to 75 years and with longer PD duration, typically around 5 years which may also contribute to disparate findings.^28,43–45^

The levels of Hg were increased in both blood and CSF of PD patients compared to controls, suggesting a potential role in PD pathophysiology. In previous studies comparing blood Hg levels in PD versus controls, one study found an increase,^46^ while three others found no differences.^47–49^ Regarding CSF Hg levels, studies found either no significant difference^29,31^ or decreased Hg in PD.^49^ The effect size of Hg difference between PD and controls in our study is small and it was likely only detectable because of the large sample size. Admittedly, the small difference in mercury concentration, approximately 0.1 µg/L in blood, is less than the regional variations documented in previous studies^50–52^ and does not significantly contribute to acute toxicity. Indeed, only one PD case and no control participants had blood Hg levels exceeding 5 μg/L - a concentration below which no adverse health effects are expected.^53^ However, given the long prodromal phase of PD, the possibility that a long-lasting, mild increase in Hg levels in body fluids may elevate PD risk or accelerate the manifestation of latent synucleinopathy cannot be excluded. Whole blood Hg concentration reflects methyl-Hg from dietary sources and elemental Hg vapor form amalgams, both of which can cross the blood-brain barrier, with brain being their target organ.^22^ This is supported by the strong correlation between blood and CSF Hg levels observed in our study. Our results thus suggest that even a small increase in blood Hg levels is accompanied by a corresponding increase in CSF, which may have implications for CNS toxicity.

Interestingly, the only longitudinal study found lower serum Hg values in the prodromal PD stage relative to controls that significantly increased in the second sample acquired 4–12 years after the PD diagnosis.^28^ The study by Petersen et al.^47^ is remarkable; authors did not find significant differences in blood Hg between PD and control groups despite identifying consumption of pilot-whale meat, a major dietary source of methyl-Hg, as a significant risk factor for developing PD in Faroe Islands inhabitants. To that point, age-adjusted incidence of PD in Faroe islands is twice as high compared to other parts of Denmark^54^ which may be theoretically linked to higher dietary intake of Hg. On the other hand, PD incidence in Korea is not remarkably high,^55^ despite the general population having higher blood Hg concentration^56^ compared to the European general population,^57^ likely due to high fish consumption. Dental amalgam fillings are other significant sources of Hg exposure, with notable disparities in their usage across countries. Interestingly, Czechia stands out for its exceptionally high utilization, with the proportion of amalgam fillings surpassing 90% of all dental fillings in 2012.^58^ In a Taiwanese study, individuals with at least one amalgam dental filling over an eight-year period were 1.6 times more likely to develop PD.^59^ PD patients were found to have significantly more amalgam fillings in another case-control study.^60^ The largest epidemiological study assessing Hg burden in the Czech population found a similar median blood Hg concentration (0.65 µg/L) as in our healthy controls, indicating that our control group represents a regional population sample with Hg levels comparable to those in other European countries.^52,57^ Notably, the association between number of amalgam fillings and Hg levels in PD has not been explored; some metallomic studies in PD even excluded participants with amalgam fillings.^29^ It is also important to note that although elevated mercury levels from dental amalgam are consistently reported, the increase is mild and has not been unequivocally linked to adverse health effects.^61^

Other research also supports the involvement of Hg exposure in PD pathophysiology.^46,62^ Additionally, a neuropathological study revealed Hg deposits in neurons and oligodendrocytes in substantia nigra, striatum, thalamus, and motor cortex of PD patients exceeding levels found in control brains.^63^

Lower levels of Cr in blood and CSF in PD observed in our study are difficult to interpret. It is not entirely clear whether Cr is purely toxic or also an essential element involved in physiological processes.^64^ Cr was suggested to enhance the action of insulin and improve carbohydrate metabolism.^65^ Interestingly, impaired insulin signaling has been implicated in the development of PD, suggesting that improving insulin sensitivity, potentially through Cr, could have some benefits.^66^ Some previous studies in PD also found low CSF Cr levels that were decreasing with disease duration and severity^29,49^ while other studies did not confirm this finding in CSF^30,31,67^ and blood.^49^ Similarly, our findings of lower CSF values of Mn, Co, V, and Ni in PD only add to heterogeneous results of previous studies^26,27,29–31,45,49,68,69^ some of which also reported lower CSF values of multiple metals in PD.^29,49^ It has been shown that Fe binds to alpha-synuclein during fibril formation.^70^ It is theoretically possible that other metals may also become trapped in pathologic alpha-synuclein aggregates, leading to their lower levels in CSF.

Blood Cd level was positively associated with the number of cigarettes per month and lower blood Cd in male PD patients was likely related to lower prevalence of smokers in the PD group. Smoking is known as the major contributor of Cd exposure in the population^23^ and also as a protective factor for PD development. Thus, detecting lower blood Cd as an epiphenomenon of lower number of smokers in the PD group is dependent on population smoking rate and proportion of smokers among included participants in a particular study. Unfortunately, previous studies comparing Cd levels in PD and controls did not report smoking status of participants.^29,44,48,49^

It is important to note that Cr, Ni, and Co are components of surgical steel alloy from which medical needles are made of. Particularly in the case of CSF, where metal concentrations are low, contamination from lumbar needles cannot be excluded. Therefore, different batches of lumbar needles as well as small alterations in the spinal tap procedure may theoretically lead to different CSF metal concentrations. In our controls, the correlations among Cr, Ni, and Co concentrations are very strong (r_s_ > 0.7) suggesting potential contamination. The tight correlation between blood and CSF concentrations of Cr along with its significantly lower blood concentration in PD argues against CSF contamination. However, differences in other metals should be interpreted with extreme caution.

The results of Fe analysis suggest abnormalities in both systemic and CNS Fe metabolism, with an intriguing discrepancy between CSF and whole blood levels. Contrary to previous findings which suggest that high serum iron and hemoglobin levels may protect against PD development,^71,72^ we observed higher whole blood Fe levels in PD compared to controls. This difference remained significant even after adjusting for hemoglobin levels. Previous studies examining serum/plasma Fe levels in PD returned highly heterogeneous results. Consequently, three meta-analyses reported decrease^32,73,74^ while other four meta-analyses reported no difference^75–78^ in serum/plasma Fe levels between PD patients and controls. Interestingly, one meta-analysis found higher blood Fe levels in PD in studies where Fe was measured in fasting subjects, as was the case in our study.^78^ This raises the possibility that PD might influence diurnal fluctuations in blood Fe concentration^79,80^ rather than total body Fe stores.

Regarding CSF, our finding of lower Fe concentration in PD is supported by some but not all previous studies. Reported CSF Fe levels in PD patients have varied, with some studies showing increases, others decreases, and some no change at all. Meta-analyses have generally found no difference in CSF Fe levels between PD patients and controls^32,73–75^ with the exception of one that reported lower CSF Fe level in PD.^78^ In PD, Fe levels in CSF were previously shown to be positively associated with disease duration.^25^ In our current study, we have found CSF concentration of Fe and other metals including Cu, Zn, Se increase with age for both, PD and controls. This suggests that differences in disease stage as well as age of participants across studies might contribute to the varying results seen in CSF Fe levels. Overall, an intimate link between Fe and PD was proven in many studies^70^ including a pharmaceutical trial in de novo untreated PD patients where conservative chelation with deferiprone led to clinical worsening compared to placebo. This was interpreted as a result of insufficient dopamine synthesis due to low Fe supply for tyrosine-hydroxylase, a rate limiting enzyme of dopamine synthesis.^81^ Given the significant heterogeneity in the literature, we speculate that Fe metabolism in PD may be disturbed at multiple levels. This disruption could manifest as either increased or decreased Fe levels in body fluids, depending on factors such as the time-of-day samples are taken, disease severity, treatment status, genetic predisposition, dietary habits, and microbiome interactions affecting gastrointestinal absorption. Analogically, excessive alcohol consumption impairs Fe metabolism in a multifactorial way and may lead to iron deficiency in some and hemochromatosis in others.^82^ Interestingly, both excessive alcohol intake and PD lead to changes in transferrin sialylation, although in a different direction.^83^

Several metals exhibited known sex-dependent variations.^84,85^ These included lower blood and CSF levels of Fe and Zn in women, likely reflecting hormonal influences and menstrual blood loss, and higher blood Cu levels in women due to estrogen-driven regulation of ceruloplasmin. Blood Pb levels were higher in men, possibly due to occupational exposure and higher hematocrit levels. While other observed differences, including lower blood Co, Mo, and Ni in women, remain unclear, they may be related to renal clearance rates or dietary variations. However, it is important to note that no significant group-by-sex interactions were observed, indicating that sex does not differentially influence the relationship between metal levels and PD pathogenesis.

Intriguingly, no clear correlations between metal concentration and clinical symptoms were observed, except for CSF Se, which correlated with the severity of autonomic symptoms in PD despite the absence of a significant between-group difference. The interpretation of this finding remains highly speculative. The early presence of autonomic symptoms in PD is associated with a more severe, so-called diffuse-malignant subtype.^86^ Increased CSF Se may, therefore, be specifically linked to this disease subtype, potentially explaining the lack of difference in the overall PD cohort. While excessive Se levels are toxic, selenium plays a crucial role in neuroprotection through selenoproteins such as glutathione peroxidases and thioredoxin reductases, which counteract oxidative stress and neuroinflammation. Additionally, selenoprotein P may regulate CSF levels of other metals, including Fe and Cu,^87^ and theoretically interfere with alpha-synuclein aggregation.^88^ However, without further Se speciation, it remains unclear whether higher Se levels contributes to pathogenesis or represent a compensatory response in alpha-synucleinopathy.

Our study has several limitations. Mainly, only a subgroup of PD patients had CSF available for analysis. Additionally, only a clinical control group could be used for comparison due to obvious obstacles in obtaining CSF from healthy volunteers. This clinical control group consisted of patients with neurological symptoms, such as sleep disorders or functional movement disorders, as well as patients undergoing elective urological surgery. While these conditions are not associated with neurodegeneration, they may influence systemic or CNS metal homeostasis and contribute to variability in the observed metal concentrations. For example, chronic sleep disturbances could theoretically impact oxidative stress pathways, functional disorders may involve altered neuroendocrine regulation, and urologic conditions may affect metal excretion. However, the heterogeneity of the control group minimizes the risk of systematic bias arising from any one condition and provides a broader baseline against which to compare PD-related changes. Therefore, while some confounding that contributes to inter-individual variability in metal concentrations through systemic mechanisms cannot be excluded, it is unlikely to account for the observed group differences. Given that plasma/serum was not analyzed in our study, we cannot determine the compartment of whole blood, plasma or intracellular, in which the alteration of metals takes place. Additionally, although we observed alterations in metal concentrations in PD, we lack data to identify potential sources of exposure, such as environmental, occupational, dietary, or other factors. Lastly, due to the heterogeneous findings in previous metallomic studies on PD, a validation cohort is necessary to confirm our results.

In conclusion, we have found increased Hg and decreased Cr concentrations in the blood and CSF of de novo treatment-naïve PD patients. Although it is at this stage not possible to ascertain whether differences in Hg and Cr concentrations are related to PD pathophysiology or are just an epiphenomenon, this finding should trigger further research on the relations between the sources of exposure and metabolism of these metals and PD. Our results support abnormal Fe regulation in blood and CSF in PD patients. Additionally, we have observed altered concentrations of Co, Mn, V, and Ni in CSF and Zn in blood in PD compared to controls. Although these findings may reflect either a cause or a consequence of PD pathology and should be interpreted with caution, they highlight the nuanced nature of PD-metals associations. Future studies should aim to replicate our findings in independent cohorts of de novo and, ideally, prodromal PD patients. In parallel, examining metal speciation - such as differentiating between inorganic and organic forms - would help clarify the biological activity and toxicity potential of the metals observed. Furthermore, longitudinal studies are needed to explore associations between trace metal concentrations and the progression of clinical features, including motor severity and fluctuations, autonomic dysfunction, and cognitive decline.

## Supplemental Material

sj-docx-1-pkn-10.1177_1877718X251367303 - Supplemental material for Blood and cerebrospinal fluid metallomics uncover mercury, chromium, and iron alterations in *de novo* Parkinson's disease Supplemental material, sj-docx-1-pkn-10.1177_1877718X251367303 for Blood and cerebrospinal fluid metallomics uncover mercury, chromium, and iron alterations in *de novo* Parkinson's disease by Petr Dušek, Ranjani Ganapathy Subramanian, Tereza Serranová, Karel Šonka, Evžen Růžička and Jan Kuta in Journal of Parkinson's Disease
