# Supplementary material for: Blood and cerebrospinal fluid metallomics uncover mercury, chromium, and iron alterations in de novo Parkinson's disease
Source: J Parkinsons Dis. 2025 Sep 4;15(8):1383–96. doi: 10.1177/1877718X251367303 (PMC13347535; doi:10.1177/1877718X251367303)
Supplement: sj-docx-1-pkn-10.1177_1877718X251367303 - Supplemental material for Blood and cerebrospinal fluid metallomics uncover mercury, chromium, and iron alterations in de novo Parkinson's disease [file sj-docx-1-pkn-10.1177_1877718X251367303.docx]

**Supplemental Material**

**Blood and cerebrospinal fluid metallomics uncover mercury, chromium, and iron alterations in de novo Parkinson’s disease**

**Supplemental Table 1.** Results of censored quantile regression at quantiles τ =0.25, 0.5, and 0.75 adjusted for age and sex

| **Metal** | **β (τ =0.25, lower quartile)** | **p** | **β (τ =0.25, median)** | **p** | **β (τ =0.75, upper quartile)** | **p** |  |
| --- | --- | --- | --- | --- | --- | --- | --- |
| **V (blood)** | -0.0000 | 0.980 ^s^ | -0.0004 | 0.842 | -0.0100 | **<0.001** ^s^ |  |
| **Ni (CSF)** | - | - | -0.1998† | **<0.001**^s^ | -0.1686 | **<0.001**^s, a+^ |  |
| **As (CSF)** | +0.0015 | 0.650 | +0.0025 | 0.757 | -0.0063 | 0.799 |  |
| **Cd (CSF)** | -0.0000 | 0.989 | -0.0003 | 0.726 | -0.0005 | 0.687 |  |
| **Pb (CSF)** | - | - | -0.0039† | 0.359 | -0.0128 | **0.044** |  |
| **Hg (CSF)** | - | - | +0.0022† | **<0.001**^s^ | +0.0020 | **<0.001**^s^ |  |
| Models were fit using censored quantile regression with the Portnoy method. Censoring was handled using a left-censored survival object based on each element's limit of detection. Note: For CSF Ni, Pb, and Hg, CQR estimation at τ = 0.25 was not feasible due to model convergence issues or excessive left-censoring and this quantile was therefore omitted from reporting.  β, estimated effect of group (Parkinson’s Disease vs. controls); †, closest feasible estimate to median under Portnoy  ^a+^ significant positive effect of age; ^a-^ significant negative effect of age; ^s^ significant effect of sex | | | | | | | |

**Supplemental Table 2.** Cross-correlation table for metal concentrations in blood for controls

|  | **V** | **Cr** | **Mn** | **Fe** | **Co** | **Ni** | **Cu** | **Zn** | **As** | **Se** | **Mo** | **Cd** | **Hg** | **Pb** |
| --- | --- | --- | --- | --- | --- | --- | --- | --- | --- | --- | --- | --- | --- | --- |
| **age** | 0.252 | 0.186 | -0.273 | -0.130 | -0.303 | -0.174 | -0.302 | -0.060 | 0.186 | -0.168 | -0.157 | 0.002 | 0.028 | 0.318 |
| **BMI** | 0.065 | -0.057 | 0.064 | 0.100 | -0.112 | -0.034 | 0.160 | 0.177 | 0.014 | -0.053 | -0.017 | -0.171 | -0.073 | 0.016 |
| **alcohol** | **0.472** | -0.133 | -0.256 | -0.275 | -0.006 | -0.150 | -0.269 | -0.244 | 0.091 | -0.130 | -0.238 | 0.038 | -0.085 | 0.230 |
| **smoking** | 0.044 | -0.123 | 0.014 | 0.051 | 0.163 | 0.087 | 0.064 | 0.037 | -0.039 | -0.142 | -0.159 | **0.606** | -0.125 | 0.005 |
| **diabetes** | 0.138 | 0.031 | -0.069 | 0.015 | 0.008 | 0.106 | -0.136 | 0.106 | 0.002 | -0.086 | -0.106 | -0.089 | -0.119 | 0.066 |
| **creatinine** | 0.105 | -0.037 | -0.173 | 0.087 | -0.300 | -0.120 | -0.271 | 0.065 | 0.195 | 0.037 | 0.098 | -0.130 | 0.051 | 0.329 |
| **hemoglobin** | 0.135 | -0.029 | -0.037 | 0.246 | -0.342 | -0.223 | -0.322 | 0.230 | 0.137 | 0.150 | -0.159 | -0.039 | 0.188 | 0.381 |
| **V** |  | 0.104 | -0.179 | -0.283 | -0.023 | -0.093 | -0.061 | -0.213 | 0.178 | -0.132 | -0.145 | 0.013 | 0.050 | 0.335 |
| **Cr** | 0.104 |  | 0.156 | 0.061 | 0.252 | **0.450** | -0.084 | -0.098 | 0.150 | -0.117 | -0.108 | 0.057 | 0.065 | 0.018 |
| **Mn** | -0.179 | 0.156 |  | **0.526** | 0.316 | 0.306 | 0.168 | **0.502** | 0.201 | 0.198 | 0.008 | 0.215 | 0.198 | 0.031 |
| **Fe** | -0.283 | 0.061 | **0.526** |  | -0.041 | 0.123 | -0.072 | **0.806** | 0.179 | 0.313 | -0.104 | 0.199 | 0.275 | 0.359 |
| **Co** | -0.023 | 0.252 | 0.316 | -0.041 |  | **0.708** | 0.402 | -0.093 | 0.052 | -0.051 | 0.166 | 0.151 | -0.052 | -0.313 |
| **Ni** | -0.093 | **0.450** | 0.306 | 0.123 | **0.708** |  | 0.280 | -0.034 | 0.097 | -0.110 | 0.073 | 0.121 | -0.050 | -0.225 |
| **Cu** | -0.061 | -0.084 | 0.168 | -0.072 | 0.402 | 0.280 |  | -0.069 | 0.041 | 0.094 | 0.219 | 0.021 | 0.003 | -0.212 |
| **Zn** | -0.213 | -0.098 | **0.502** | **0.806** | -0.093 | -0.034 | -0.069 |  | 0.023 | 0.265 | -0.100 | 0.222 | 0.162 | 0.352 |
| **As** | 0.178 | 0.150 | 0.201 | 0.179 | 0.052 | 0.097 | 0.041 | 0.023 |  | 0.238 | 0.042 | 0.046 | **0.606** | 0.240 |
| **Se** | -0.132 | -0.117 | 0.198 | 0.313 | -0.051 | -0.110 | 0.094 | 0.265 | 0.238 |  | 0.339 | -0.168 | 0.328 | -0.009 |
| **Mo** | -0.145 | -0.108 | 0.008 | -0.104 | 0.166 | 0.073 | 0.219 | -0.100 | 0.042 | 0.339 |  | -0.281 | 0.035 | -0.231 |
| **Cd** | 0.013 | 0.057 | 0.215 | 0.199 | 0.151 | 0.121 | 0.021 | 0.222 | 0.046 | -0.168 | -0.281 |  | -0.158 | 0.257 |
| **Hg** | 0.050 | 0.065 | 0.198 | 0.275 | -0.052 | -0.050 | 0.003 | 0.162 | **0.606** | 0.328 | 0.035 | -0.158 |  | 0.125 |
| **Pb** | 0.335 | 0.018 | 0.031 | 0.359 | -0.313 | -0.225 | -0.212 | 0.352 | 0.240 | -0.009 | -0.231 | 0.257 | 0.125 |  |
| Spearman correlation coefficients (rho) are shown, significant (p<0.05) positive and negative associations are marked in blue and red color respectively whereby the saturation reflects strength of the relationship; rho > 0.4 are marked by bold font. | | | | | | | | | | | | | | |

**Supplemental Table 3.** Cross-correlation table for metal concentrations in blood for PD

|  | **V** | **Cr** | **Mn** | **Fe** | **Co** | **Ni** | **Cu** | **Zn** | **As** | **Se** | **Mo** | **Cd** | **Hg** | **Pb** |  |
| --- | --- | --- | --- | --- | --- | --- | --- | --- | --- | --- | --- | --- | --- | --- | --- |
| **age** | 0.066 | 0.082 | -0.117 | -0.117 | -0.095 | 0.097 | 0.005 | -0.063 | -0.005 | -0.279 | 0.264 | 0.192 | -0.205 | 0.216 |  |
| **BMI** | -0.005 | 0.222 | -0.120 | -0.051 | 0.041 | 0.155 | -0.041 | -0.127 | -0.088 | -0.199 | -0.081 | -0.230 | -0.231 | -0.089 |  |
| **alcohol** | 0.244 | -0.014 | -0.086 | 0.266 | 0.021 | -0.045 | -0.214 | 0.228 | 0.161 | 0.051 | **-0.496** | -0.120 | 0.131 | **0.420** |  |
| **smoking** | 0.041 | 0.020 | 0.098 | -0.013 | 0.007 | -0.040 | 0.098 | 0.004 | -0.050 | 0.077 | -0.074 | 0.339 | 0.074 | 0.006 |  |
| **diabetes** | 0.045 | 0.177 | -0.008 | -0.113 | -0.046 | -0.016 | -0.211 | -0.106 | 0.031 | 0.038 | -0.097 | -0.068 | -0.151 | 0.076 |  |
| **creatinine** | 0.202 | 0.009 | -0.026 | 0.258 | -0.115 | -0.059 | -0.242 | 0.249 | 0.092 | 0.025 | -0.092 | -0.252 | 0.049 | 0.207 |  |
| **hemoglobin** | 0.045 | 0.037 | 0.057 | **0.545** | -0.027 | -0.089 | -0.316 | 0.333 | 0.012 | 0.058 | -0.314 | -0.196 | 0.121 | 0.236 |  |
| **MDS-UPDRS III** | -0.067 | -0.178 | -0.021 | -0.020 | -0.019 | -0.002 | 0.114 | -0.014 | -0.192 | -0.042 | 0.025 | -0.030 | -0.254 | -0.057 |  |
| **SCOPA-AUT** | -0.112 | -0.002 | 0.054 | -0.074 | 0.089 | 0.038 | 0.078 | -0.009 | -0.086 | -0.115 | 0.125 | 0.076 | -0.180 | 0.017 |  |
| **MoCA** | -0.052 | 0.196 | -0.066 | 0.052 | 0.050 | 0.014 | 0.072 | -0.056 | 0.202 | 0.062 | -0.046 | -0.133 | 0.234 | -0.066 |  |
| **UPSIT** | 0.021 | 0.162 | 0.002 | -0.062 | 0.202 | -0.037 | 0.142 | -0.111 | 0.052 | 0.183 | -0.069 | -0.102 | 0.103 | -0.234 |  |
| **V** |  | 0.061 | -0.145 | -0.023 | 0.003 | 0.044 | 0.030 | -0.001 | 0.027 | 0.078 | -0.152 | -0.112 | 0.019 | 0.242 |  |
| **Cr** | 0.061 |  | -0.248 | -0.398 | -0.030 | 0.240 | -0.297 | **-0.407** | 0.029 | -0.194 | -0.012 | -0.225 | -0.164 | -0.298 |  |
| **Mn** | -0.145 | -0.248 |  | 0.356 | 0.131 | -0.188 | 0.182 | 0.286 | -0.090 | 0.088 | 0.021 | 0.399 | 0.062 | 0.165 |  |
| **Fe** | -0.023 | -0.398 | 0.356 |  | -0.036 | -0.335 | -0.005 | **0.746** | -0.010 | 0.161 | -0.291 | 0.193 | 0.184 | **0.555** |  |
| **Co** | 0.003 | -0.030 | 0.131 | -0.036 |  | 0.356 | 0.207 | -0.035 | 0.057 | 0.151 | 0.019 | 0.112 | -0.056 | -0.086 |  |
| **Ni** | 0.044 | 0.240 | -0.188 | -0.335 | 0.356 |  | 0.007 | -0.229 | 0.091 | -0.245 | 0.092 | -0.072 | -0.137 | -0.242 |  |
| **Cu** | 0.030 | -0.297 | 0.182 | -0.005 | 0.207 | 0.007 |  | 0.046 | 0.014 | 0.143 | 0.177 | 0.290 | -0.107 | 0.177 |  |
| **Zn** | -0.001 | **-0.407** | 0.286 | **0.746** | -0.035 | -0.229 | 0.046 |  | 0.011 | 0.121 | -0.280 | 0.181 | 0.123 | **0.420** |  |
| **As** | 0.027 | 0.029 | -0.090 | -0.010 | 0.057 | 0.091 | 0.014 | 0.011 |  | 0.300 | -0.089 | 0.061 | **0.415** | 0.164 |  |
| **Se** | 0.078 | -0.194 | 0.088 | 0.161 | 0.151 | -0.245 | 0.143 | 0.121 | 0.300 |  | -0.179 | 0.022 | 0.272 | 0.122 |  |
| **Mo** | -0.152 | -0.012 | 0.021 | -0.291 | 0.019 | 0.092 | 0.177 | -0.280 | -0.089 | -0.179 |  | 0.088 | -0.135 | -0.152 |  |
| **Cd** | -0.112 | -0.225 | 0.399 | 0.193 | 0.112 | -0.072 | 0.290 | 0.181 | 0.061 | 0.022 | 0.088 |  | 0.110 | 0.193 |  |
| **Hg** | 0.019 | -0.164 | 0.062 | 0.184 | -0.056 | -0.137 | -0.107 | 0.123 | **0.415** | 0.272 | -0.135 | 0.110 |  | 0.147 |  |
| **Pb** | 0.242 | -0.298 | 0.165 | **0.555** | -0.086 | -0.242 | 0.177 | **0.420** | 0.164 | 0.122 | -0.152 | 0.193 | 0.147 |  |  |
| Spearman correlation coefficients (rho) are shown, significant (p<0.05) positive and negative associations are marked in blue and red color respectively whereby the saturation reflects strength of the relationship; rho > 0.4 are marked by bold font.  MDS-UPDRS III, Movement Disorder Society-Unified Parkinson's Disease Rating Scale part III; SCOPA-AUT, Scale for Outcomes in Parkinson's disease for Autonomic symptoms; MoCA, Montreal Cognitive Assessment; UPSIT, University of Pennsylvania Smell Identification Test | | | | | | | | | | | | | | | |

**Supplemental Table 4.** Cross-correlation table for metal concentrations in CSF for controls

|  | **V** | **Cr** | **Mn** | **Fe** | **Co** | **Ni** | **Cu** | **Zn** | **As** | **Se** | **Mo** | **Cd** | **Hg** | **Pb** |
| --- | --- | --- | --- | --- | --- | --- | --- | --- | --- | --- | --- | --- | --- | --- |
| **age** | 0.090 | 0.377 | 0.007 | **0.583** | 0.286 | 0.397 | **0.535** | **0.556** | 0.395 | 0.285 | -0.031 | 0.182 | 0.156 | 0.097 |
| **BMI** | -0.380 | -0.062 | -0.294 | -0.008 | -0.072 | 0.030 | 0.108 | 0.058 | -0.002 | -0.106 | -0.210 | -0.107 | -0.132 | -0.155 |
| **alcohol** | -0.137 | -0.053 | -0.066 | 0.242 | 0.115 | -0.035 | 0.354 | 0.354 | 0.385 | **0.601** | 0.233 | 0.405 | 0.020 | -0.069 |
| **smoking** | -0.054 | 0.146 | -0.078 | 0.068 | 0.141 | 0.133 | 0.130 | 0.043 | 0.082 | -0.206 | 0.077 | -0.088 | 0.133 | 0.157 |
| **diabetes** | 0.144 | 0.236 | 0.189 | 0.393 | 0.350 | 0.344 | **0.429** | 0.370 | 0.158 | 0.293 | 0.105 | 0.011 | -0.263 | -0.018 |
| **creatinine** | -0.262 | 0.186 | -0.179 | 0.242 | -0.043 | 0.167 | 0.120 | 0.081 | 0.260 | -0.096 | 0.066 | 0.125 | **0.412** | 0.145 |
| **hemoglobin** | -0.126 | -0.153 | -0.029 | 0.086 | -0.178 | -0.072 | -0.147 | 0.000 | -0.046 | -0.209 | 0.029 | -0.097 | 0.109 | -0.172 |
| **V** |  | **0.442** | **0.673** | 0.394 | **0.419** | **0.480** | 0.237 | **0.452** | 0.081 | 0.287 | 0.049 | 0.280 | -0.038 | **0.491** |
| **Cr** | **0.442** |  | **0.475** | **0.688** | **0.704** | **0.804** | **0.500** | **0.537** | 0.307 | 0.261 | 0.104 | 0.256 | 0.195 | 0.284 |
| **Mn** | **0.673** | **0.475** |  | **0.464** | **0.520** | **0.520** | 0.250 | 0.360 | 0.155 | 0.164 | 0.221 | 0.198 | -0.019 | 0.366 |
| **Fe** | 0.394 | **0.688** | **0.464** |  | **0.644** | **0.745** | **0.726** | **0.763** | **0.428** | **0.413** | 0.204 | 0.341 | 0.229 | 0.287 |
| **Co** | **0.419** | **0.704** | **0.520** | **0.644** |  | **0.824** | **0.575** | **0.595** | 0.327 | 0.355 | 0.187 | 0.199 | 0.115 | 0.108 |
| **Ni** | **0.480** | **0.804** | **0.520** | **0.745** | **0.824** |  | **0.546** | **0.630** | 0.213 | 0.185 | 0.208 | 0.365 | 0.104 | 0.262 |
| **Cu** | 0.237 | **0.500** | **0.250** | **0.726** | **0.575** | **0.546** |  | **0.721** | **0.461** | **0.626** | 0.287 | 0.257 | 0.086 | 0.130 |
| **Zn** | **0.452** | **0.537** | **0.360** | **0.763** | **0.595** | **0.630** | 0.721 |  | **0.405** | **0.449** | 0.160 | 0.294 | 0.131 | 0.334 |
| **As** | 0.081 | 0.307 | 0.155 | **0.428** | 0.327 | 0.213 | **0.461** | **0.405** |  | 0.307 | 0.084 | -0.015 | 0.245 | 0.109 |
| **Se** | 0.287 | 0.261 | 0.164 | **0.413** | 0.355 | 0.185 | **0.626** | **0.449** | 0.307 |  | 0.253 | 0.215 | 0.046 | 0.111 |
| **Mo** | 0.049 | 0.104 | 0.221 | 0.204 | 0.187 | 0.208 | 0.287 | 0.160 | 0.084 | 0.253 |  | -0.042 | 0.063 | 0.067 |
| **Cd** | 0.280 | 0.256 | 0.198 | 0.341 | 0.199 | 0.365 | 0.257 | 0.294 | -0.015 | 0.215 | -0.042 |  | 0.114 | 0.217 |
| **Hg** | -0.038 | 0.195 | -0.019 | 0.229 | 0.115 | 0.104 | 0.086 | 0.131 | 0.245 | 0.046 | 0.063 | 0.114 |  | 0.059 |
| **Pb** | **0.491** | 0.284 | 0.366 | 0.287 | 0.108 | 0.262 | 0.130 | 0.334 | 0.109 | 0.111 | 0.067 | 0.217 | 0.059 |  |
| Spearman correlation coefficients (rho) are shown, significant (p<0.05) positive and negative associations are marked in blue and red color respectively whereby the saturation reflects strength of the relationship; rho > 0.4 are marked by bold font. | | | | | | | | | | | | | | |

**Supplemental Table 5.** Cross-correlation table for metal concentrations in CSF for PD

|  | **V** | **Cr** | **Mn** | **Fe** | **Co** | **Ni** | **Cu** | **Zn** | **As** | **Se** | **Mo** | **Cd** | **Hg** | **Pb** |
| --- | --- | --- | --- | --- | --- | --- | --- | --- | --- | --- | --- | --- | --- | --- |
| **age** | -0.031 | 0.123 | 0.304 | **0.404** | 0.000 | 0.012 | 0.398 | 0.329 | 0.077 | 0.319 | 0.230 | 0.141 | 0.041 | 0.141 |
| **BMI** | 0.175 | -0.070 | -0.297 | 0.104 | -0.211 | -0.228 | 0.211 | 0.254 | 0.097 | 0.276 | 0.138 | 0.114 | 0.006 | 0.114 |
| **alcohol** | 0.080 | 0.040 | -0.112 | 0.058 | 0.050 | -0.158 | -0.113 | 0.068 | 0.068 | -0.056 | -0.288 | 0.005 | -0.013 | -0.036 |
| **smoking** | 0.061 | 0.046 | -0.058 | 0.020 | 0.016 | -0.077 | 0.076 | -0.029 | 0.007 | -0.088 | -0.050 | -0.196 | 0.137 | 0.066 |
| **diabetes** | 0.249 | 0.075 | 0.218 | 0.320 | -0.068 | -0.168 | 0.333 | 0.340 | 0.184 | 0.208 | -0.013 | 0.082 | -0.002 | 0.293 |
| **creatinine** | -0.010 | -0.102 | -0.234 | 0.070 | -0.107 | 0.036 | -0.072 | 0.137 | 0.006 | 0.058 | 0.029 | 0.053 | -0.099 | -0.093 |
| **hemoglobin** | 0.074 | -0.056 | -0.311 | -0.025 | -0.066 | 0.083 | -0.164 | 0.080 | -0.016 | -0.092 | -0.086 | -0.152 | -0.023 | -0.114 |
| **MDS-UPDRS III** | -0.134 | 0.001 | 0.091 | -0.054 | -0.017 | 0.062 | -0.008 | -0.007 | 0.006 | -0.211 | 0.117 | 0.047 | 0.020 | -0.124 |
| **SCOPA-AUT** | -0.222 | -0.170 | 0.076 | 0.315 | 0.218 | 0.081 | 0.375 | 0.236 | 0.119 | **0.423** | 0.158 | 0.253 | -0.023 | -0.189 |
| **MoCA** | 0.055 | -0.086 | -0.204 | 0.057 | -0.123 | -0.016 | 0.134 | 0.018 | 0.155 | 0.101 | -0.040 | 0.030 | 0.171 | 0.091 |
| **UPSIT** | -0.010 | -0.129 | -0.181 | -0.129 | -0.066 | -0.265 | -0.039 | -0.036 | 0.116 | -0.066 | -0.097 | -0.180 | 0.065 | 0.029 |
| **V** |  | **0.585** | 0.288 | 0.324 | -0.089 | -0.103 | 0.146 | 0.347 | 0.157 | 0.143 | -0.055 | 0.275 | 0.115 | **0.772** |
| **Cr** | **0.585** |  | **0.479** | **0.501** | 0.307 | 0.244 | 0.204 | 0.363 | 0.164 | 0.187 | -0.008 | 0.228 | 0.232 | **0.608** |
| **Mn** | 0.288 | **0.479** |  | **0.413** | **0.439** | 0.173 | 0.300 | 0.251 | 0.168 | 0.199 | 0.066 | 0.195 | 0.195 | 0.378 |
| **Fe** | 0.324 | **0.501** | **0.413** |  | 0.359 | 0.075 | **0.805** | **0.777** | 0.261 | **0.730** | 0.134 | 0.280 | 0.384 | 0.375 |
| **Co** | -0.089 | 0.307 | **0.439** | 0.359 |  | **0.472** | 0.259 | 0.225 | 0.187 | 0.253 | -0.013 | -0.035 | 0.096 | 0.000 |
| **Ni** | -0.103 | 0.244 | 0.173 | 0.075 | **0.472** |  | -0.096 | -0.093 | -0.098 | -0.109 | 0.057 | 0.030 | -0.131 | -0.184 |
| **Cu** | 0.146 | 0.204 | 0.300 | **0.805** | 0.259 | -0.096 |  | **0.742** | 0.107 | **0.790** | 0.213 | 0.062 | 0.319 | 0.272 |
| **Zn** | 0.347 | 0.363 | 0.251 | **0.777** | 0.225 | -0.093 | **0.742** |  | 0.208 | **0.683** | 0.081 | 0.214 | 0.248 | **0.483** |
| **As** | 0.157 | 0.164 | 0.168 | 0.261 | 0.187 | -0.098 | 0.107 | 0.208 |  | 0.092 | -0.240 | 0.223 | 0.286 | 0.196 |
| **Se** | 0.143 | 0.187 | 0.199 | **0.730** | 0.253 | -0.109 | **0.790** | **0.683** | 0.092 |  | 0.160 | 0.150 | 0.230 | 0.292 |
| **Mo** | -0.055 | -0.008 | 0.066 | 0.134 | -0.013 | 0.057 | 0.213 | 0.081 | -0.240 | 0.160 |  | 0.003 | -0.028 | 0.001 |
| **Cd** | 0.275 | 0.228 | 0.195 | 0.280 | -0.035 | 0.030 | 0.062 | 0.214 | 0.223 | 0.150 | 0.003 |  | 0.289 | 0.157 |
| **Hg** | 0.115 | 0.232 | 0.195 | 0.384 | 0.096 | -0.131 | 0.319 | 0.248 | 0.286 | 0.230 | -0.028 | 0.289 |  | 0.196 |
| **Pb** | **0.772** | **0.608** | 0.378 | 0.375 | 0.000 | -0.184 | 0.272 | **0.483** | 0.196 | 0.292 | 0.001 | 0.157 | 0.196 |  |
| Spearman correlation coefficients (rho) are shown, significant (p<0.05) positive and negative associations are marked in blue and red color respectively whereby the saturation reflects strength of the relationship; rho > 0.4 are marked by bold font.  MDS-UPDRS III, Movement Disorder Society-Unified Parkinson's Disease Rating Scale part III; SCOPA-AUT, Scale for Outcomes in Parkinson's disease for Autonomic symptoms; MoCA, Montreal Cognitive Assessment; UPSIT, University of Pennsylvania Smell Identification Test | | | | | | | | | | | | | | |

**Supplemental Table 6.** Correlations between blood and CSF concentrations of metals for PD and controls

|  | **V** | **Cr** | **Mn** | **Fe** | **Co** | **Ni** | **Cu** | **Zn** | **As** | **Se** | **Mo** | **Cd** | **Hg** | **Pb** |  |
| --- | --- | --- | --- | --- | --- | --- | --- | --- | --- | --- | --- | --- | --- | --- | --- |
| **PD** | 0.187 | **0.323** | 0.158 | -0.224 | 0.155 | **-0.282** | -0.012 | -0.124 | **0.664** | 0.186 | **0.620** | -0.175 | **0.630** | **-0.330** |  |
| **controls** | 0.003 | **0.441** | 0.093 | 0.178 | 0.243 | **0.282** | -0.042 | -0.002 | **0.833** | 0.01 | **0.446** | -0.033 | **0.689** | -0.096 |  |
| Spearman correlation coefficients (rho) are shown, significant (p<0.05) associations are marked by bold font. | | | | | | | | | | | | | | | |

**Supplemental Table 7.** Blood/CSF metal concentration ratio

| **Metal** | **PD** | **Controls** | **unadjusted p** |  |
| --- | --- | --- | --- | --- |
| **Fe^1^** | M 36,158 (12,528)  F 42,274 (20,808) | M 29,704 (12,950)  F 38,953 (12,916) | **<0.001**^s,a-^ |  |
| **Zn^1^** | 557.5 (152.4) | 564.2 (164.6) | 0.497 ^a-^ |  |
| **Cu^1^** | M 77.2 (22.0)  F 90.3 (25.5) | M 70.6 (24.3)  F 94.4 (21.2) | 0.337 ^s,a-^ |  |
| **Se^1^** | M 79.6 (20.0)  F 85.9 (24.7) | M 73.7 (23.9)  F 69.4 (24.6) | M 0.440 ^a-^  **F 0.007 ^a-^** |  |
| **Mn^1^** | 21.0 (9.5) | 17.4 (7.9) | 0.016 ^a-^ |  |
| **Mo^1^** | M 3.7 (1.1)  F 4.4 (1.3) | M 3.5 (0.9)  F 4.5 (1.7) | 0.591 ^s^ |  |
| **Cr^1^** | 5.8 (5.1) | 3.5 (2.4) | **0.004** |  |
| **Co^1^** | 26.9 (15.2) | 19.9 (12.0) | **0.010 ^a-^** |  |
| **V^2^** | 1.1 (1.0; 0.5 – 1.2) | 0.8 (0.7; 0.3-1.1) | **0.011** |  |
| **Ni^2^** | 20.5 (11.0; 3.9-18.5) | 9.5 (4.7; 2.4-9.8) | **0.003** |  |
| **As^2^** | 24.8 (20.0; 15.0-30.5) | 20.1 (18.0; 14.5-23.0) | 0.146 |  |
| **Cd^2^** | 92.3 (52.5; 27.9-115.0) | 147.3 (70.0; 40.0-253.8) | 0.030 |  |
| **Hg^2^** | 197.1 (200.0; 113.3-261.7) | 225.3 (193.3; 139.1-293.3) | 0.439 |  |
| **Pb^2^** | 2462 (2280; 543-4230) | 2262 (1106; 405-3360) | 0.357 |  |
| ^1^ values are reported as mean (standard deviation); statistical analysis performed using ANCOVA with sex and age as covariates.  ^2^ values are reported as mean (median; interquartile range); statistical analysis performed using Mann-Whitney U test.  ^s^ significant effect of sex; in case of significant sex effect, values are reported for males and females separately.  ^a+^ significant positive effect of age; ^a-^ significant negative effect of age.  Significant (p<0.05) between-group differences are marked by bold font.  PD: Parkinson disease; CSF: cerebrospinal fluid; M: male; F: female. | | | | |
